# Differential Brain Activation to Angry Faces by Elite Warfighters: Neural Processing Evidence for Enhanced Threat Detection

**DOI:** 10.1371/journal.pone.0010096

**Published:** 2010-04-14

**Authors:** Martin P. Paulus, Alan N. Simmons, Summer N. Fitzpatrick, Eric G. Potterat, Karl F. Van Orden, James Bauman, Judith L. Swain

**Affiliations:** 1 University of California San Diego, San Diego, California, United States of America; 2 Naval Health Research Center, San Diego, California, United States of America; 3 United States Olympic Training Center, Chula Vista, California, United States of America; 4 Naval Special Warfare Center, San Diego, California, United States of America; 5 OptiBrain Consortium, San Diego, California, United States of America; 6 Singapore Institute for Clinical Sciences-A*STAR and National University of Singapore, Singapore, Singapore; University of Groningen, Netherlands

## Abstract

**Background:**

Little is known about the neural basis of elite performers and their optimal performance in extreme environments. The purpose of this study was to examine brain processing differences between elite warfighters and comparison subjects in brain structures that are important for emotion processing and interoception.

**Methodology/Principal Findings:**

Navy Sea, Air, and Land Forces (SEALs) while off duty (n = 11) were compared with n = 23 healthy male volunteers while performing a simple emotion face-processing task during functional magnetic resonance imaging. Irrespective of the target emotion, elite warfighters relative to comparison subjects showed relatively greater right-sided insula, but attenuated left-sided insula, activation. Navy SEALs showed selectively greater activation to angry target faces relative to fearful or happy target faces bilaterally in the insula. This was not accounted for by contrasting positive versus negative emotions. Finally, these individuals also showed slower response latencies to fearful and happy target faces than did comparison subjects.

**Conclusions/Significance:**

These findings support the hypothesis that elite warfighters deploy greater processing resources toward potential threat-related facial expressions and reduced processing resources to non-threat-related facial expressions. Moreover, rather than expending more effort in general, elite warfighters show more focused neural and performance tuning. In other words, greater neural processing resources are directed toward threat stimuli and processing resources are conserved when facing a nonthreat stimulus situation.

## Introduction

Extreme environments are characterized as those situations that place a high demand on the physiological, affective, cognitive, and/or social processing resources of the individual. Optimal performance in extreme environments is a complex process and its neural basis is poorly understood. There is a surging interest in the use of neuroscience approaches to examine and possibly improve performance in military personnel [1]. Several investigators have examined warfighters in extreme environments to better understand impairments of optimal performance. For example, Lieberman and colleagues [2] examined the effects of sleep deprivation and environmental stress on performance and mood in Navy Sea, Air, and Land Forces (SEALs) and found significant behavioral decrements. More recently, Morgan and collaborators [3] proposed a specific mechanism that may contribute to maintenance of optimal performance in extreme environments. Specifically, these authors suggested that vagal suppression, which is modulated by the right insular cortex [4], is associated with enhanced performance under high-stress conditions.

Optimal performance in extreme situations is a complex problem that is affected by multiple factors [5], ranging from genetic differences to interpersonal variables. One approach to examining the factors contributing to optimal performance is to compare groups of individuals who are considered “optimal performers” based on special skill sets or training with healthy volunteers. Although, there are currently no experimental probes that have been studied extensively to examine “optimal” performance per se, one can begin to delineate the neural processes that differentiate these groups. The development of a neural signature of elite performers is a first step towards understanding the brain processing characteristics of these indivudals. In particular, there may not be a simple increase or decrease in neural response or behavioral performance, but a capacity to adjust neural processing and behavioral performance during a task to most efficaciously match the environmental demands.

We recently proposed that maintaining an interoceptive balance in the presence of significant perturbations may be a neural marker of optimal performance [5]. Interoception can be defined as the sense of the internal body state and includes a range of sensations, such as pain [6], temperature [7], itch [8], tickle [9], sensual touch [10], [11], muscle tension [12], air hunger [13], stomach pH [14], and intestinal tension [15]. Taken together, these sensations provide an integrated sense of the body's physiological condition [16]. Thus, the interoceptive system plays a crucial role in maintaining a homeostatic state under extreme perturbations. It provides body-related information to other brain areas that monitor value or salience, is important for evaluating reward, and provides critical input to cognitive control processes. This approach is based on extensive work by Craig [17], Critchley [18], and others [19], [20] that has provided new insights into how the interoceptive system modulates self-monitoring and creates urges to act to maintain homeostasis. In particular, several neural substrates are thought to mediate these processes, which include the insular cortex in processing emotion-related tasks and the anterior cingulate as a link to cognitive control processes.

In this study, we sought to determine whether elite warfighters (i.e., SEALs), who can be considered considered an example of optimal performers in extreme environments, exhibit distinct neural processing patterns that are consistent with the notion of altered interoceptive processing. To that end, we examined off-duty Navy SEALs while performing a simple emotion face-processing task during functional magnetic resonance imaging (fMRI), and compared them with healthy male volunteers. We examined whether these elite warfighters respond distinctly to target faces exhibiting a variety of emotions. The results demonstrated that active-duty Navy SEALs exhibit a distinct pattern of brain activation during an emotion face-processing task within neural substrates that are important for interoception, indicating that elite warfighters show measurable processing differences compared with normal volunteers.

## Results

### Behavioral Results

**Figure 1:**
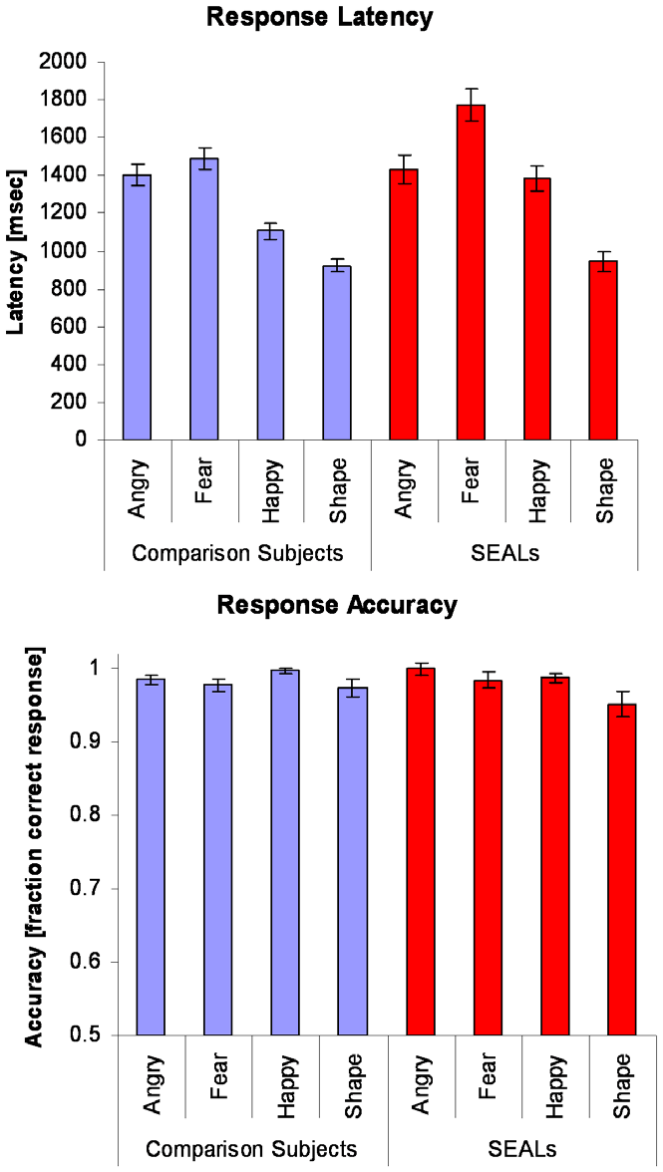
Behavioral Performance during Face Processing Task Behavioral performance on the emotion face processing task showed no differences on accuracy but subtle response latency differences across groups (see text for details).

The latency to respond to a target varied by the type of the target face, *F*(3,29) = 3.94, *p* = 0.018 (Figure 1). Although Navy SEALs did not differ from healthy male comparison subjects on the overall response latency, *F*(1,31) = 2.87, *p* = 0.10, there was a significant group-by-face interaction, *F*(3,29) = 6.21, *p* = 0.002. Specifically, Navy SEALs were relatively slower to respond to happy, *t*(32) = 3.43, *p* = 0.002 and fearful, *t*(32) = 2.74, *p* = 0.01, faces. There were no significant differences across task conditions on accuracy of responding, *F*(3,29) = 0.539, *p* = 0.659. Moreover, Navy SEALs did not differ from healthy male comparison subjects on response accuracy, *F*(1,31) = 0.714, *p* = 0.405, or on accuracy as a function of target face, *F*(3,29) = 1.14, *p* = 0.349. Taken together, although there were significant latency differences, which were primarily due to longer latencies when matching to happy or fearful target faces by the SEAL group, there were no accuracy differences across groups.

### Task-Related Activation

**Figure 2:**
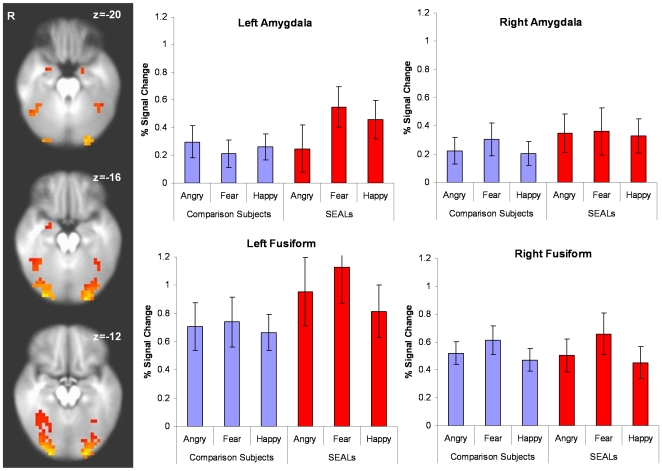
Task-related Activation Task-related brain activation in bilateral amygdala and fusiform gyrus showed no significant group differences.

Activation during the emotion face assessment task involved both limbic and paralimbic structures including bilateral insula, amygdala, and the fusiform gyrus (see Figure 2). There were no significant differences across groups in the left amygdala, *F*(1,32) = 1.42, *p* = 0.242 or in the group by target face interaction, *F*(2,63) = 2.71, *p* = 0.074. Moreover, both groups showed similar activation in the right amygdala, *F*(1,31) = 0.40, *p* = 0.529 and did not differ across target faces, *F*(2,63) = 0.16, *p* = 0.851.

### Group Differences

**Figure 3:**
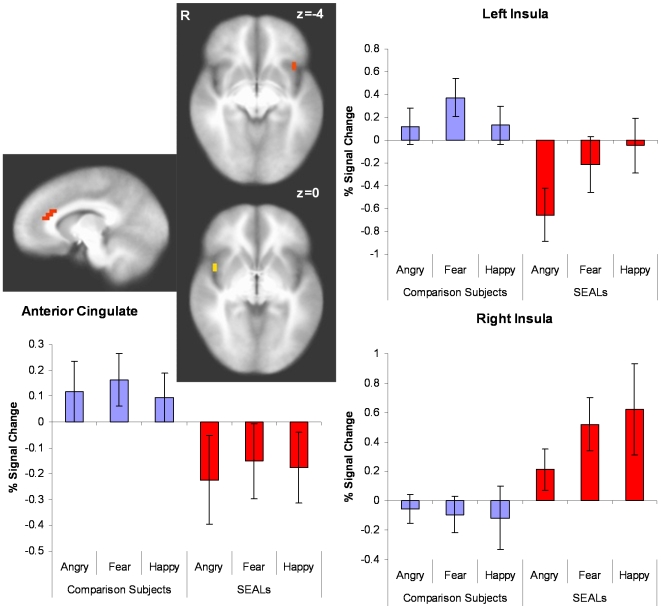
Group Differences Overall group differences showed relatively greater right insula activation in SEALs versus left insula activation in comparison subjects.

Task-related activation differed significantly across groups in three areas. Healthy volunteers, relative to SEALs, showed greater face emotion processing related activation in the left anterior insula, *F*(1,32) = 8.82, *p* = 0.005. In comparison, SEALs showed greater right mid-insula activation to faces, *F*(1,32) = 6.55, *p* = 0.015 (see Figure 3). Finally, whereas comparison subjects showed significant activation in dorsal anterior cingulate, SEALs showed relative deactivation in this area, *F*(1,32) = 11.21, *p* = 0.002. Thus, although there were no differences in task-related activation in the amygdala, SEALs showed relatively stronger right insular versus left insular activation, whereas normal volunteers showed the opposite pattern.

### Group-by Face-Interactions

**Figure 4:**
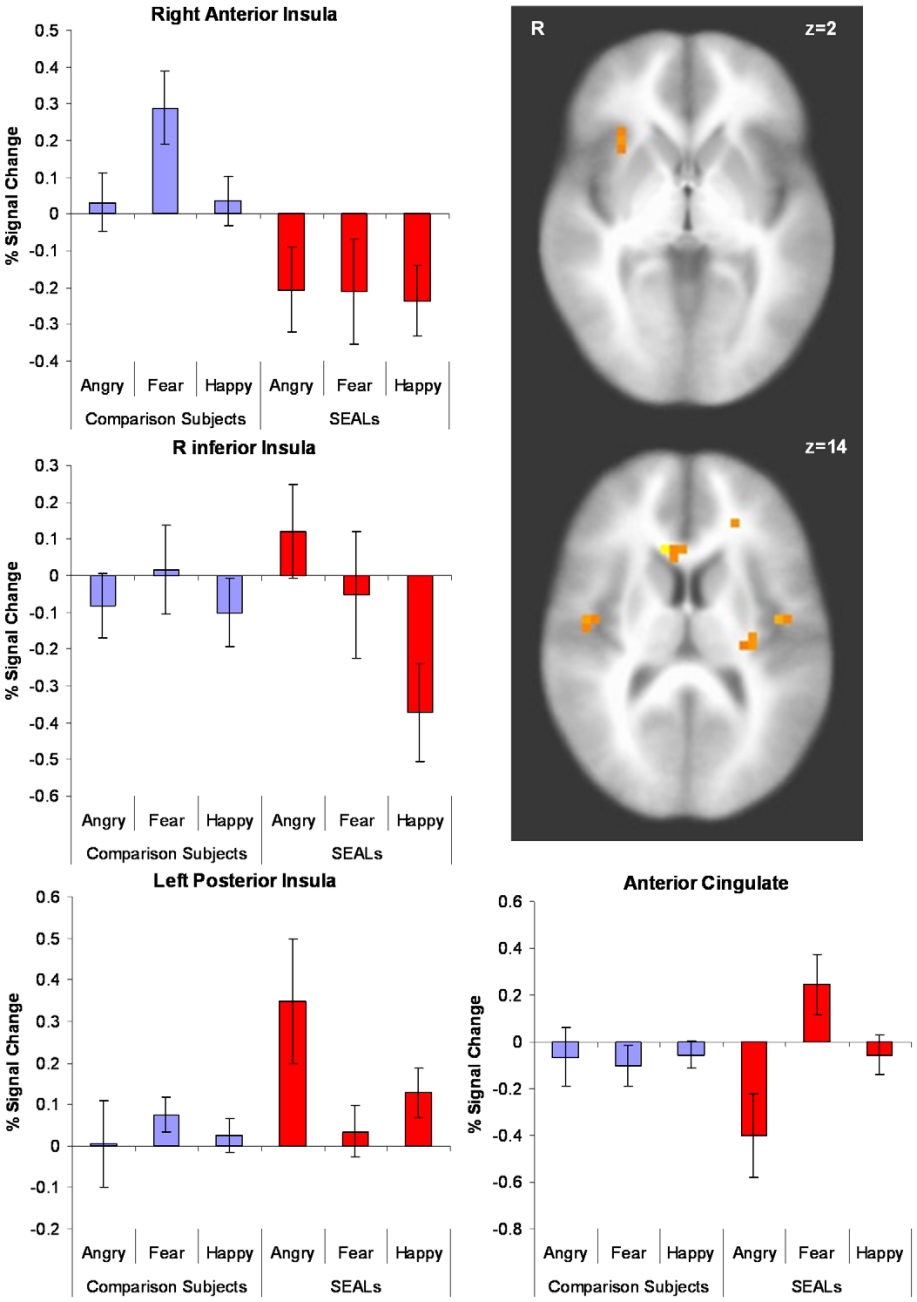
Group by Face Interactions Group-by-target face interaction revealed significantly greater activation to angry target faces, particularly in the right insular cortex.

There were two areas within the left, *F*(2,63) = 5.51, *p* = 0.006, and a trend in the right, *F*(2,63) = 2.453, *p* = 0.094, insular cortex that showed group-by-face interactions (see Figure 4). Interestingly, in the right insular cortex, SEALs showed significant activation to angry target faces relative to fearful targets, *t*(10) = 2.21, *p* = 0.05, and happy targets, *t*(10) = 2.51, *p* = 0.03, whereas the direct comparison was not significant in the left insular cortex. In comparison, normal volunteers did not show this differential effect. Two additional analyses were conducted to determine whether this was simply a positive versus negative emotional valence effect or an effect specific to anger as a target emotion (see Text S1). A reduced mixed model was computed separately for positive versus negative valenced target emotion and for anger versus fear/happiness target emotion, respectively. The group-by-emotion type interaction showed a larger area on bilateral posterior insula for anger versus fear/happiness (Figure S1) but not for positive versus negatively valenced target emotions (Figure S2).

### Brain–Behavior Relationships

There were no significant correlations across or within groups between the degree of activation in the right insular cortex during angry faces and response latency or response accuracy.

## Discussion

There are three main findings in this study. First, elite warfighters relative to comparison subjects showed relatively greater right-sided insula, but attenuated left-sided insula, activation. Second, these individuals showed selectively greater activation to angry target faces relative to fearful or happy target faces bilaterally in the insula. Third, these individuals also showed slower response latencies to fearful and happy target faces. Taken together, these findings support the notion that elite warfighters, when examined cross-sectionally, deploy greater neural processing resources toward potential threat-related facial expressions and reduced processing resources to non-threat-related facial expressions. This finding suggests that rather than expending more effort in general, elite warfighters show more focused neural and performance tuning, such that greater neural processing resources are directed toward threat stimuli and processing resources are conserved when facing a nonthreat stimulus situation. Moreover, the suggestion of relatively greater right-sided insula activation is consistent with the lateralization of feelings hypothesis, which suggests that right-sided processing is a more energy-consuming (sympathetic) condition [21].

Navy SEALs are a unique group of elite warfighters. A recently conducted systematic review [22] and qualitative assessment [23] revealed several factors that influence the degree to which individuals successfully complete Basic Underwater Demolition/SEAL (BUD/S) training, which is considered one of the most challenging military training programs. Individuals who are likely to complete this training program are characterized by an attitude of mental toughness, achievement motivation, physical strength, physical endurance, emotional stability, and team orientation. These factors are clearly multidimensional but support the critical importance of optimal monitoring and balancing of the relationship between brain processing and body functioning. These factors are reminiscent of Damasio's somatic marker model [24], which extends the James Lang theory of emotion, and involves the insular cortex that can instantiate body sensation without necessarily receiving peripheral inputs. Specifically, the somatic marker model proposes that “body states” that have been experienced during the past are instantiated in decision-making situations with uncertain outcomes, and provide weights in favor or against choosing specific options. This model has been extended by Craig [17] who suggested that body states undergo a complex integration within the insular cortex, which is critical for the process of awareness itself. Therefore, the relative neural activation differences between SEALs and comparison subjects may reflect somatic marker differences that are instantiated when presented with specific emotional faces in general and angry faces in particular.

The insula (reviewed elsewhere [25], [26]) is a paralimbic structure that constitutes the invaginated portion of the cerebral cortex, forming the base of the sylvian fissure, and is considered limbic sensory cortex by some [27]. Activation of the insular cortex has been reported in a number of processes, including pain [28], interoceptive [29], emotion-related [30], [31], cognitive [32], and social processes [33]. Moreover, we have shown that the insular cortex is an important structure for processing the anticipation of aversive emotional states [34]–[36], risk-taking [37], and decision-making [38]. In reward-related processes, the insular cortex is important for subjective feeling states and interoceptive awareness [16], [29] and together with middle and inferior frontal gyri, frontal limbic areas, and the inferior parietal lobe plays an important role in inhibitory processing [39]. Thus, differential activations in the insular cortex when assessing an emotional face could be attributed to the degree to which individuals integrate the presentation of a facial expression with the experience of other processes, such as interoception, pain, and social interactions.

Several investigators have proposed that different types of emotions are lateralized to the left- or right-sided hemisphere [21], [40], [41]. In particular, these researchers have argued that aversive, negative, or energy-consuming emotions are more right-lateralized, whereas approach, positive, or energy-saving emotions are left-lateralized. Although this assumption has been called into question or has been refined [30], [42], this notion still provides a useful heuristic for the current findings. Thus, the relatively greater right insular cortex activation by Navy SEALs supports the idea that these individuals deploy more processing resources to the potential aversive or negative affective associations with facial expressions. Moreover, together with the selectively increased activation to angry target faces in Navy SEALs, these individuals may selectively processing facial features that are critical for potentially aversive or negative consequences. It is important to point out, however, that this cross-sectional study cannot be used to differentiate what could be a trait characteristic or whether this is anger-related processing difference is a consequence of training.

We have proposed a neuroanatomical processing model as a heuristic guide to understand how one can link optimal performance to how the individual “feels inside.” This model focuses on the notion of a body prediction error (i.e., the difference between the value of the anticipated/predicted state and the value of the current interoceptive state) and consists of four components. First, information from peripheral receptors ascends via two different pathways, the A-beta-fiber discriminative pathway that conveys precise information about the “what” and “where” of the stimulus impinging on the body, and the C-fiber pathway that conveys spatially and time-integrated affective information [43]. These afferents converge via several way stations to the sensory cortex and the posterior insular cortex to provide a sense of the current body state. Second, centrally generated interoceptive states (e.g., via contextual associations from memory) reach the insular cortex via temporal and parietal cortex to generate body states based on conditioned associations [44], [45]. Third, in the insular cortex there is a dorsal-posterior to inferior-anterior organization from granular to agranular, which provides an increasingly “contextualized” representation of the interoceptive state [46], irrespective of whether it is generated internally or via the periphery. These interoceptive states are made available to the orbitofrontal cortex for context-dependent valuation [47], [48] and to the anterior cingulate cortex for error processing [49], [50] and action valuation [51], [52]. Fourth, bidirectional connections to the basolateral amygdala [26], [53], [54] and the striatum [55], particularly ventral striatum [56], provide the circuitry to calculate a body prediction error (similar to reward prediction error [57]–[59]), and provide a neural signal for salience and learning. The insular cortex relays information to other brain systems to initiate motivated action to achieve a steady state [43] by minimizing the body state prediction error. Thus, the greater activation to angry faces in SEALs may represent a relatively stronger body prediction error signal, which would help guide individuals to deploy cognitive and behavioral resources to adjust to anticipated aversive outcomes.

This investigation had several limitations. First, the group of elite warfighters we studied was relatively small and thus there could have been a significant lack of power to detect additional behavioral/functional relationships. With larger number of subjects and different tasks, other important relationships may become apparent. Second, there were no significant correlations between performance on the task and brain activation. This is not surprising, however, because this task is not design to probe emotional or cognitive processes in a performance-related manner. Future investigations will need to use performance-based paradigms (e.g., the detection of mild threat using morphed faces). Third, and most importantly, this cross-sectional study could not address the question whether the observed processing differences were part of the preexisting characteristics of individuals who were selected and then trained to become elite warfighters, or whether these neural processing differences were a consequence of training. Thus, future studies will need to examine, in a within-subjects study design, individuals prior to and again after elite warfighter training.

This study is a first step in elucidating the neural processes that characterize optimal performers. A key difference between optimal performers and comparison subjects revealed in this study is that both neural response and behavioral response are adapted such that greater resources are expended in threat-relevant conditions and conserved in nonrelevant conditions. Thus, the capacity of optimal performers to deploy resources effectively may ensure that they can perform better in extreme situations. However, more studies are needed to examine how modulation of brain resource deployment when engaging in different cognitive and affective processes contributes to optimal performance. Moreover, there is a need to examine the link between behavioral performance during a challenging cognitive or emotion-processing task, and brain-related activation, to more conclusively determine whether differential brain processing patterns directly relate to measurable behavioral performance differences. Nevertheless, this study shows that with a relatively small group of subjects one can begin to delineate the neural circuitry that contributes to performance differences. The ultimate goal of these studies is to better understand the role of these circuits in determining performance, and then to develop more targeted training interventions that will further improve individual and team performance in extreme and complex environments.

## Materials and Methods

### Participants

This study was approved by the University of California San Diego (UCSD) Institutional Review Board and all subjects signed informed consent. Subjects were recruited as healthy volunteers or as comparison subjects for studies with Afghan and Iraqi war veterans as part of the research effort supported by the Center of Excellence for Stress and Mental Health (CESAMH). All subjects were interviewed with a structured diagnostic interview (SCID) [60], modified to enable us to document the presence of posttraumatic stress disorder. Only subjects who did not have a Diagnostic and Statistical Manual of Mental Disorders DSM-IV [61] diagnosis were included in this study. Thirty-four male subjects completed the study. Specifically, 11 Navy SEALs aged 26.8 years (SD = 3.7), who all had been deployed an average of 2.8 times (range 1–5) and 23 healthy male volunteers aged 24.6 years (SD = 7.4) with 13.7 (SD = 1.6) and 12.5 (SD = 0.7) years of education participated in the study. The groups did not differ in age, *t*(32) = 0.95, *p* = 0.35, but the healthy volunteers had more years of education, *t*(32) = 2.58, *p* = 0.014. Thus, all analyses were covaried for years of education. All subjects were trained to perform the emotion face-processing task prior to testing during fMRI scanning and received $50 for participation. No restrictions were placed on the consumption of caffeinated beverages; none of the subjects were smokers.

### Task

During fMRI, each subject was tested on a slightly modified [62] version of the emotion face-processing task [63], [64]. During each 5-second trial, a subject was presented with a target face (on the top of the computer screen) and two probe faces (on the bottom of the screen) and was instructed to match the probe with the same emotional expression to the target by pressing the left or right key on a button box. A block consists of 6 consecutive trials where the target face is angry, fearful, or happy. During the sensorimotor control task, subjects were presented with 5-second trials of either vertical or horizontal ovals or circles in an analogous configuration and instructed to match the shape of the probe to the target. Each block of faces and of the sensorimotor control task was presented three times in a pseudo-randomized order. A fixation cross lasting 8 seconds was interspersed between each block presented at the beginning and end of the task (resulting in 14 fixation periods). For each trial, response accuracy and reaction time data were obtained. There were 18 trials (3 blocks of 6 trials) for each face set as well as for shapes, and the whole task lasted 512 seconds.

### Analysis

#### Acquisition of images

All scans were performed on a 3T GE CXK4 Magnet (General Electric Medical. Systems, Milwaukee, WI) at the UCSD Keck Imaging Center, which is equipped with 8 high-bandwidth receivers that allow for shorter readout times and reduced signal distortions and ventromedial signal dropout. Each 1-hour session consisted of a 3-plane scout scan (10 seconds), a standard anatomical protocol (i.e., a sagittally acquired spoiled gradient recalled sequence) (FOV = 25 cm, matrix  = 192×256, 172 sagittally acquired slices 1-mm thick, TR  = 8 ms, TE  = 3 ms, flip angle  = 12°). We used an 8-channel brain array coil to axially acquire T2*-weighted echo-planar images (EPIs) with the following parameters: FOV  = 23 cm, matrix  = 64×64, 30 slices 2.6-mm thick, gap  = 1.4 mm, TR  = 2000 ms, TE  = 32 ms, flip angle  = 90°.

#### Image analysis pathway

The basic structural and functional image processing were conducted with the Analysis of Functional NeuroImages (AFNI) software package [65]. A multivariate regressor approach detailed below was used to relate changes in EPI intensity to differences in task characteristics [66]. Echoplanar images were coregistered using a 3D-coregistration algorithm [67] that has been developed to minimize the amount of image translation and rotation relative to all other images. Six motion parameters were obtained for each subject. Three of these motion parameters were used as regressors to adjust for EPI intensity changes due to motion artifacts. All slices of the EPI scans were temporally aligned following registration to ensure that different relationships with the regressors were not due to the acquisition of different slices at different times during the repetition interval.

#### Multiple regressor analyses

The four orthogonal regressors of interest were (1) happy, (2) angry, (3) fearful, and (4) circle/oval (i.e., shape) sensorimotor condition. These 0–1 regressors were convolved with a gamma variate function [68] modeling a prototypical hemodynamic response (6–8 second delay [69]) and to account for the temporal dynamics of the hemodynamic response (typically 12–16 seconds) [70]. The convolved time series was normalized and used as a regressor of interest. A series of regressors of interest and the motion regressors were entered into the AFNI program *3DDeconvolve* to determine the height of each regressor for each subject. The main dependent measure was the voxel-wise normalized relative signal change (or percent signal change for short), which was obtained by dividing the regressor coefficient by the zero-order regressor. Spatially smoothed (4-mm full-width half-maximum Gaussian filter) percent signal change data were transformed into Talairach coordinates based on the anatomical magnetic resonance images, which was transformed manually in AFNI.

#### Anatomically constrained functional regions of interest [71]

For the amygdala region of interest, a priori regions of interest were defined by the Talairach Daemon atlas [72] and functional neuroimage analyses were constrained to the a priori defined regions of interest. For the insular cortex we extended this approach to use a probability mask. Briefly, to extract a mask for the insular cortex, we used Individual Brain Atlases using Statistical Parametric Mapping software (IBASPM, http://www.thomaskoenig.ch/Lester/ibaspm.htm), a toolbox for segmenting structural MR images. All programs in this toolbox are developed in MATLAB (http://www.mathworks.com), based on a widely used neuroimaging software package, SPM (Wellcome Trust Centre for Neuroimaging, London, UK). This package uses the nonlinear registration and gray matter segmentation processes performed through SPM5 subroutines. Three principal elements for the labeling process are used: gray matter segmentation, normalization transform matrix, which maps voxels from individual space to standardized space, and MaxPro MNI Atlas. Data from a set of an existing set of 39 individuals, with similar sociodemographic characteristics as the target population, were processed using the SPM-based voxel-based morphometry approach [73]. These data were subsequently processed using the IBASPM toolbox to obtain estimates of each individual's insula. The group insula mask was obtained by averaging across the individual insular masks and requiring that the insula voxels covered at least 50% of all subjects' gray matter.

### Statistics

All second-level analyses were conducted using the statistical programming language R (http://cran.r-project.org/) and with SPSS software, version 10.0 [74]. Specifically, a mixed-model analysis was conducted with the R program *lme*, which is part of the *nlme* library. The fixed effects were emotion type, group, education, and response latency; the random effects were subjects (i.e., an individual intercept was fitted for each subject). Moreover, we conducted voxel-wise multiple linear regression analyses with performance on the emotion-processing task (latency to respond to angry, fearful, or happy faces) as independent measures, and the percent signal change between faces and the sensorimotor control condition as the dependent measure using the *lm* program of R.

## Supporting Information

Text S1 This file provides supporting information.
(0.04 MB DOC)

Figure S1 A reduced linear mixed effects model focusing on anger-related processing revealed significant group differences in bilateral posterior insula.
(1.93 MB TIF)

Figure S2 A reduced linear mixed effects model focusing on valence differences revealed significant group differences in bilateral insula and ventral ACC.
(1.87 MB TIF)
