# Supplementary material for: Differential Brain Activation to Angry Faces by Elite Warfighters: Neural Processing Evidence for Enhanced Threat Detection
Source: PLoS One. 2010 Apr 14;5(4):e10096. doi: 10.1371/journal.pone.0010096 (PMC2854680; doi:10.1371/journal.pone.0010096)
Supplement: Text S1 — This file provides supporting information. (0.04 MB DOC) [file pone.0010096.s001.doc]

**Supporting Information**

Two additional analyses were conducted with a reduced design matrix using the linear mixed-effects model. First, a design matrix was constructed to contrast anger – fear/happiness. Second, another design matrix was constructed to contrast anger/fear – happiness. The goal was to determine whether the insular cortex showed a differential sensitivity to the former but not to the latter in SEALs versus healthy comparison subjects.

The first analysis revealed a large area in bilateral posterior insula that showed a significant group-by-emotion type interaction: left insula, *F*(1,65) = 7.80, *p* = 0.007; right insula, *F*(1,65) = 5.38, *p* = 0.023. Moreover, the group-by-emotion type interaction identified by the main analysis (e.g., in the left posterior insula), was significant with the reduced analysis using the contrast anger – fear/happiness *F*(1,65) = 8.94, *p* = 0.004, but not using the anger/fear – happiness contrast, *F*(1,65) = 0.15, *p* = 0.697

Figure S1 shows the group-by-face interactions for the reduced model focusing on anger – fear/happiness in the bilateral insular cortex. In comparison, Figure S2 shows the reduced model fear/anger – happiness. Comparing these images, one can clearly delineate that bilateral insular cortex shows a group-by-face interaction for the former contrast but not for the latter.

Figure Legends for Supporting Information

**Figure S1**.

Title: Anger-related Activation Differences

A reduced linear mixed effects model focusing on anger-related processing revealed significant group differences in bilateral posterior insula.

**Figure S2**.

Title: Negatively versus Positively valenced Emotion Activation Differences

A reduced linear mixed effects model focusing on valence differences revealed significant group differences in bilateral insula and ventral ACC.
