# Supplementary figures and images for: Differential Brain Activation to Angry Faces by Elite Warfighters: Neural Processing Evidence for Enhanced Threat Detection
Source: PLoS One. 2010 Apr 14;5(4):e10096. doi: 10.1371/journal.pone.0010096 (PMC2854680; doi:10.1371/journal.pone.0010096)

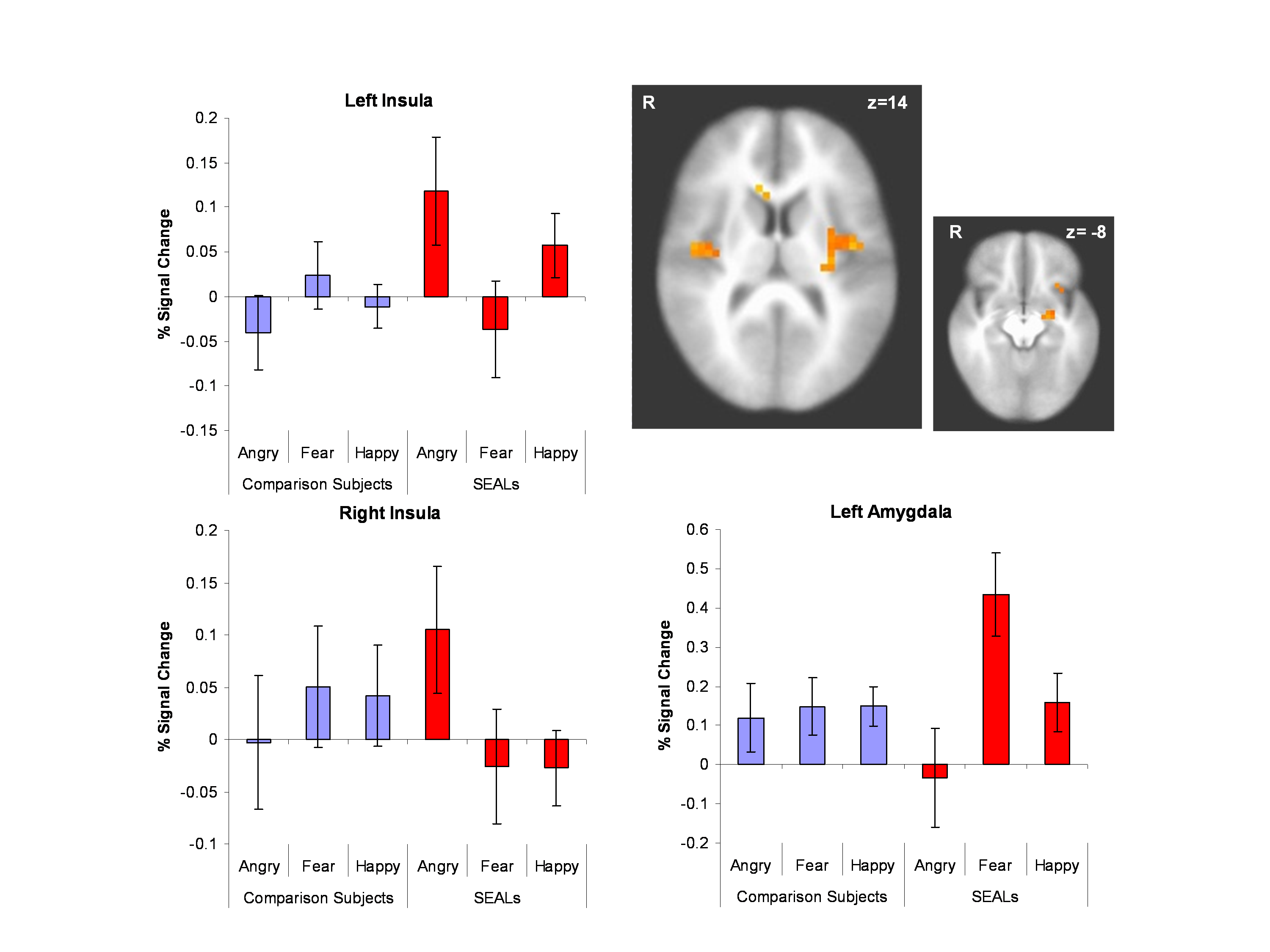

Supplement: Figure S1 — A reduced linear mixed effects model focusing on anger-related processing revealed significant group differences in bilateral posterior insula. (1.93 MB TIF) [file pone.0010096.s002.tif]

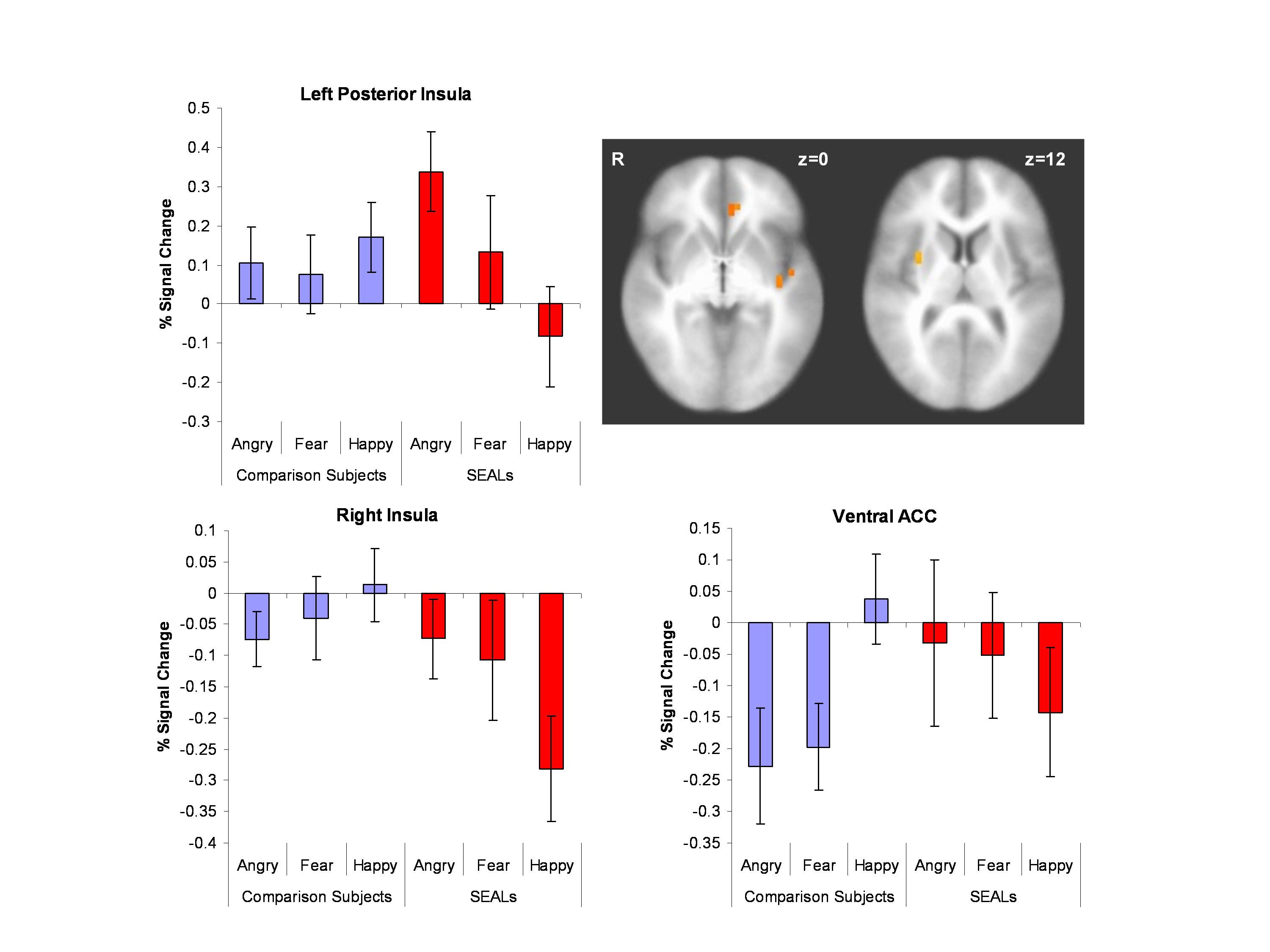

Supplement: Figure S2 — A reduced linear mixed effects model focusing on valence differences revealed significant group differences in bilateral insula and ventral ACC. (1.87 MB TIF) [file pone.0010096.s003.tif]
